# Low Concentrations of Hydrogen Peroxide or Nitrite Induced of *Paracoccidioides brasiliensis* Cell Proliferation in a Ras-Dependent Manner

**DOI:** 10.1371/journal.pone.0069590

**Published:** 2013-07-29

**Authors:** Ana Eliza Coronel Janu Haniu, Juliana Terzi Maricato, Pedro Paulo Moraes Mathias, Daniele Gonçalves Castilho, Rodrigo Bernardi Miguel, Hugo Pequeno Monteiro, Rosana Puccia, Wagner Luiz Batista

**Affiliations:** 1 Departamento de Microbiologia, Imunologia e Parasitologia - Universidade Federal de São Paulo, São Paulo, Brazil; 2 Departamento de Bioquímica/Biologia Molecular – Centro de Terapia Celular e Molecular (CTCMol), Universidade Federal de São Paulo, São Paulo, Brazil; 3 Departamento de Ciências Biológicas - Universidade Federal de São Paulo/Campus Diadema, São Paulo, Brazil; Geisel School of Medicine at Dartmouth, United States of America

## Abstract

*Paracoccidioides brasiliensis*, a causative agent of paracoccidioidomycosis (PCM), should be able to adapt to dramatic environmental changes inside the infected host after inhalation of air-borne conidia and transition to pathogenic yeasts. Proteins with antioxidant functions may protect fungal cells against reactive oxygen (ROS) and nitrogen (RNS) species generated by phagocytic cells, thus acting as potential virulence factors. Ras GTPases are involved in stress responses, cell morphology, and differentiation in a range of organisms. Ras, in its activated form, interacts with effector proteins and can initiate a kinase cascade. In lower eukaryotes, Byr2 kinase represents a Ras target. The present study investigated the role of Ras in *P. brasiliensis* after *in vitro* stimulus with ROS or RNS. We have demonstrated that low concentrations of H_2_O_2_ (0.1 mM) or NO_2_ (0.1–0.25 µM) stimulated *P. brasiliensis* yeast cell proliferation and that was not observed when yeast cells were pre-incubated with farnesyltransferase inhibitor. We constructed an expression plasmid containing the Byr2 Ras-binding domain (RBD) fused with GST (RBD-Byr2-GST) to detect the Ras active form. After stimulation with low concentrations of H_2_O_2_ or NO_2_, the Ras active form was observed in fungal extracts. Besides, NO_2_ induced a rapid increase in *S*-nitrosylated Ras levels. This alternative posttranslational modification of Ras, probably in residue Cys123, would lead to an exchange of GDP for GTP and consequent GTPase activation in *P. brasiliensis*. In conclusion, low concentrations of H_2_O_2_ or NO_2_ stimulated *P. brasiliensis* proliferation through Ras activation.

## Introduction

*Paracoccidioides brasiliensis* is a thermo-dependent dimorphic fungus responsible for paracoccidioidomycosis (PCM), a systemic mycosis that is prevalent in Latin America. The major protective host immune response against *P. brasiliensis* is mediated by cells, as evidenced by granuloma formation [1]. The interaction between *P. brasiliensis* and alveolar macrophages is a crucial step for the establishment and progression of infection in susceptible hosts. The yeast pathogenic phase of *P. brasiliensis* is a facultative intracellular pathogen that is able to survive and replicate within the phagosome of inactivated murine and human macrophages [2]. By contrast, macrophages can engulf this microorganism and confine it within phagosomes where, by action of microbicidal molecules and/or by restriction of essential nutrients, the pathogen can be destroyed. Among the molecules that exert fungicidal action in phagosomes are hydrogen peroxide (H_2_O_2_), nitric oxide (NO) and their derivatives. These are generated by NADPH oxidase and inducible nitric oxide synthase (iNOS), respectively [3], [4]. Immune system cells activate the NADPH oxidase complex to generate radical superoxide (O_2_^−^), which is subsequently converted into H_2_O_2_. Macrophages express iNOS with activation of the L-arginine-nitric oxide pathway, and subsequent NO production.

The ability of pathogenic fungi to cause disease is related to their ability to survive in the host. Many microorganisms evolved strategies to survive in hostile conditions, like those within phagocytic cells. While studying *P. brasiliensis* transcriptional responses after internalization by murine macrophages, Tavares et al. [5] showed an increase in the expression of genes encoding antioxidant molecules like SOD, and proteins like Hsp60 involved in thermal stress. This work has clearly demonstrated that the parasite has antioxidant mechanisms and responds to oxidative and nitrosative stress [5]. Evidence has increased to support the idea that reactive oxygen species (ROS) and reactive nitrogen species (RNS) may have an important role as regulators of signal transduction, being able to participate in signaling pathways associated with physiological and pathophysiological processes [6], [7].

Signaling pathways that control morphological changes, cell proliferation and stress response in *P. brasiliensis* are largely unknown, but in other dimorphic fungi the involvement of cAMP (cyclic adenosine 3′, 5′-monophosphate) and MAPK (mitogen-activated protein kinase) is known to be an important factor in this process [8]. Modulation of MAP kinases is an important and highly conserved event in eukaryotes. It is composed of a series of protein kinases, which can sequentially phosphorylate other proteins so that the signal transmissions from the point of origin (typically the cell membrane) to the nucleus can occur [9]. As a result, the target molecules (including transcription factors) are phosphorylated [10], [11]. In this context, the Ras protein is prominent in the regulation of signal transduction pathways that mediate adaptive changes. Ras belongs to a large family of low molecular-weight proteins (21 kDa) with GTPase activity. Ras GTPases are molecular switches that are active when GTP-bound and inactive when GDP-bound. Both processes are regulated by enzymatic reactions. Guanine nucleotide exchange factors (GEFs) catalyze the release of GDP from the guanine nucleotide-binding pocket, mediating the exchange of GDP for GTP. The activation state of Ras is self-limited by its intrinsic GTPase activity, which is enhanced to critical regulatory levels by GTPase-activating enzymes (GAPs) [12]. Ras is involved in signal transduction pathways connecting events from many cell surface receptors to intracellular processes [13]. In mammals, depending on the cellular context, Ras activation can stimulate cell division cycle, morphogenesis, differentiation, or apoptosis [13].

In microorganisms, the Ras protein is similarly involved in growth and development processes, morphological changes, and stress responses. In *Saccharomyces cerevisiae*, *Cryptococcus neoformans*, and *Aspergillus fumigatus*, among other fungi, two Ras isoforms (Ras1 and Ras2) have been identified [8]. Waugh et al. [14] demonstrated that *C. neoformans* Ras proteins share some degree of functional redundancy, since both Ras1 and Ras2 mutants were viable and phenotypically similar to wild type. In *Trichoderma reesei*, both Ras1 and Ras2 play similar roles in morphogenesis and adjusting cAMP level, but Ras2 is also involved in regulation of cellulase gene expression [15]. Differential functions of Ras1 and Ras2 were also described in *Beauveria bassiana* [16]. In *Schizosaccharomyces pombe* only Ras1 was identified, which controls two different downstream signaling pathways [17]. However, endomembrane Ras activates a Cdc42 pathway to mediate cell polarity, while plasma membrane Ras selectively regulates a MAP kinase pathway to mediate mating pheromone signalling [18].

Ras proteins are conserved at the *N*-termini, but differ substantially at the *C*-termini, where 10–20 amino acids form the hypervariable region. Ras proteins are anchored to the membranes by a series of post-translational modifications occurring at the *C*-terminus. Anchoring of Ras in membranes is believed to be absolutely required for biological activity. Ras proteins contain a CAAX motif at the *C*-terminus (C = cysteine, A = aliphatic amino acid, and X = any amino acid), where farnesyl transferase-mediated cysteine farnesylation occurs in the cytosol. This posttranslational modification prompts Ras association with the endoplasmatic reticulum (ER). Farnesylation is followed by the cleavage of the three *C*-terminal residues (AAX) and subsequent carboxymethylation of the farnesyl-cysteine [19], [20].

In *P. brasiliensis*, two Ras isoforms were characterized with important roles during fungal dimorphism, thermal stress, and in parasite-host interactions [21]. The prenylation site (CAAX) was detected in both isoforms, but with variable sequences (CVIM in Ras1 and CLIL in Ras2) [21]. Nevertheless, nothing is known about GTPases functions in *P. brasiliensis* for adaptive responses to oxidative and nitrosative stress. In the present study, we investigated the importance of *P. brasiliensis* Ras GTPase after *in vitro* stimulation with ROS and RNS. Using a novel probe that detects activated Ras (Ras-GTP), we showed that low concentrations of NO and H_2_O_2_ mediate cell signaling triggered with the participation of Ras, leading to cell proliferation in *P. brasiliensis*. Furthermore, we showed that Ras is *S*-nitrosylated in our test conditions. Therefore, this work shows the beneficial role of Ras activation by low levels of ROS and RNS in *P. brasiliensis* cell proliferation.

## Materials and Methods

### 2.1. Fungal strain and growth conditions

We used *P. brasiliensis*, isolate Pb18, in our experiments. Unless otherwise mentioned, cells were cultured and maintained at 37°C in modified YPD medium (0.5% yeast extract, 0.5% casein peptone, and 1.5% glucose, pH 6.5). CFU count was performed in supplemented BHI plates (Becton Dickinson Company) containing 4% fetal calf serum, 5% spent medium, ampicillin (100 IU/mL) and streptomycin (100 µg/mL).

### 2.2. NO and H_2_O_2_ stimulation and quantification of P. brasiliensis CFU

In experiments involving oxidative and nitrosative stress, *P. brasiliensis* cells were cultivated in modified YPD for 5 days at 37°C. Yeast cells (1×10^5^) were seeded in a 6-well culture plate subjected to a 24-h period of starvation with F12 medium, to reduce or stop fungal growth until starting the treatment. This strategy was used to verify the role of H_2_O_2_ or NO_2_ stimulus on fungal growth and cell signaling. Yeast cells were exposed to different concentrations of H_2_O_2_ or NaNO_2_ (in culture medium mildly acidic, pH 5.5; in this condition NaNO_2_ releases NO), for 5 h at 37°C [22], [23], [24]. Then yeast cells were washed and incubated for 24 h at 37°C under shaking in fresh culture media. Finally, 100 µL were plated in supplemented BHI plates for 7 days at 37°C. The experiment was repeated three times. Cell proliferation was evaluated by colony formation unit counts (CFU).

Growth curves were performed by evaluating fungal counts during different days of growth. For that, yeast cell suspension aliquots (100 µl) were stained with equal volumes of Trypan Blue vital dye and counted in a Neubauer chamber (for 4, 8 and 12 days), where viable cells did not stain by the vital dye.

### 2.3. Plasmid construction

The Ras Binding Domain (RBD) coding sequence of *P. brasiliensis* Byr2 kinase (GenBank accession number EEH46080, Ste11 *S. cereviseae* homolog) was obtained by PCR, as described previously [25]. Briefly, the RBD region was synthetized by using the primer sense, 5′ CCCTTCCTCCAAATTGGCC 3′, containing an *Eco*RI site, and the downstream primer anti-sense, 5′ GTGGCTGTCTAATGTT 3′, bearing a *Xho*I site. The PCR fragment generated was cloned using the pJET vector kit (Fermentas). The RBD region (502 bp) was obtained by *Eco*RI and *Xho*I restriction and cloned into a pGEX-4T2 vector (GE Healthcare) in same restriction sites. DNA sequencing confirmed the open reading frame in the expression vector. The expression of RBD(Byr2)-GST fusion protein in *E. coli* was induced with 1 mM 1-thio-β-D-galactopyranoiside (IPTG) for 3 h at 30°C and the fusion protein was purified in glutathione-Sepharose beads. The beads were washed with PBS containing protease and phosphatase inhibitors, suspended in PBS containing 10% glycerol and protease inhibitors, and stored at −80°C.

### 2.4. Ras activation in P. brasiliensis

Ras activation was determined using the RBD(Byr2)-GST fusion protein, which tightly binds to the GTP-associated Ras form. After stimulus, yeast cells were collected by centrifugation, washed (3 times), and disrupted with glass beads in ice-cold lysis buffer (25 mM HEPES, pH 7.5, 150 mM NaCl, 1% (w/v) Nonidet P-40, 0.25% (w/v) sodium deoxycholate, 10% (w/v) glycerol, 25 mM NaF, 10 mM MgCl_2_, 1 mM EDTA, 1 mM sodium vanadate, one tablet of Protease Inhibitor, Roche Diagnostic, Mannheim, Germany, in 50 mL of extraction medium). The solubilized extract was centrifuged at 14,000× *g* for 15 min, and the supernatant was used in pull-down assays. The protein content of the cell extract was determined with Bradford reagent (Bio-Rad, Hercules, CA, USA). A protein sample (1 mg) was incubated with glutathione-Sepharose beads associated with RBD(Byr2)-GST for 3 h with gentle rocking. The samples were spun at 7,200× *g* for 10–20 sec, and the resin was washed three times with lysis/binding/wash buffer (7,200× *g* for 30 sec). The final pull-down was assayed by Western blot probed with mouse monoclonal anti-Ras antibody (Oncogene, Research Products). The remaining lysate was probed with the same antibody to determine the levels of total and endogenous Ras. The ratio between Ras signal intensity bound to RBD(Byr2)-GST beads and that obtained from total Ras, determined by densitometry, is proportional to Ras activity [26], [27].

A control for probe RBD(Byr2)-GST specificity was performed as described Colombo et al. [27], with some modifications. Pb18 total extracts (1 mg) were incubated in PBS containing protease inhibitor (one tablet of Protease Inhibitor, Roche Diagnostic, Mannheim, Germany, in 50 mL of extraction medium) with 1 mM GTP (Sigma) or GDP (Sigma) at room temperature for 1 h with gentle rocking. Samples (bound either to GTP or to GDP) were incubated with glutathione-Sepharose beads containing cross-linked RBD(Byr2)-GST for 3 h with gentle rocking and detected by Western blotting using anti-Ras antibodies.

### 2.5. Western blotting

For Western blotting, proteins (25–50 µg) were separated in 10 or 12% polyacrylamide gels and transferred to nitrocellulose membranes. After blocking, the membranes were incubated overnight at 4°C with primary anti-Ras antibody. A secondary antibody (anti-mouse) conjugated with horseradish peroxidase was used in the second step of the procedure (room temperature incubation for 1 h). Immunoblots were developed using the Super Signal® (Pierce, Rockford, USA) system.

### 2.6. Detection of Ras S-nitrosylation

The biotin switch technique (BST) was performed to detect Ras *S*-nitrosylation, as described by Forrester et al. [28]. To detect Ras *S*-nitrosylation in *P. brasiliensis* after H_2_O_2_ or nitrite treatment, yeasts were cultured in F12 medium for 24 h (starvation), then treated with H_2_O_2_ or nitrite for increasing time periods, and then yeasts were disrupted with glass beads in buffer containing 25 mM HEPES, 50 mM NaCl, 0.1 mM EDTA, 1% NP-40, 0.5 mM PMSF, and protease inhibitors (Roche Diagnostic, Mannheim, Germany), pH 7.4. Cell debris was removed by centrifugation, and samples (1 – 0.6 mg protein extract) were diluted to 1.8 mL with HEN buffer (100 mM Hepes, 1 mM EDTA, 0.1 mM neocuproine, pH 8.0); SDS and MMTS were added to final concentrations of 2.5 and 0.1%, respectively. Following frequent vortex and incubation at 50°C in the dark for 20 min, lysates were precipitated with 3 volumes of acetone at −20°C for 1 h. Proteins were centrifuged at 2,000× *g* for 15 min, and the protein pellet was gently washed with 70% acetone (four times). The pellets were suspended in 240 µL HENS (HEN buffer added 1% SDS). Samples were further incubated with 30 µL biotin-HPDP (2.5 mg/ml) in the presence or absence of 20 mM ascorbate at room temperature, in the dark for 1 h. After acetone precipitation, proteins were resuspended in 250 µL HENS, followed by addition of 750 µL of neutralization buffer (25 mM HEPES, 100 mM NaCl, 1 mM EDTA, 0.5% Triton X-100, pH 7.5). Fifty microliters of streptavidin-agarose beads (pre-washed) were added to each sample and incubated overnight at 4°C. Beads were washed with washing buffer (neutralization buffer with 600 mM NaCl) four times. To detect *S*-nitrosylated proteins, 50 µL of 2× SDS sample buffer were added to the beads and tested by immunoblotting with anti-Ras antibody.

### 2.7. Statistical analysis

Data are expressed as means ± SD. The statistical analysis of significance was assessed by one-way analysis of variance using the Student's *t*-test for comparison. *p*<0.05 was considered statistically significant.

## Results

### 3.1. Low concentrations of ROS and RNS induced cell proliferation in P. brasiliensis

**Figure 1:**
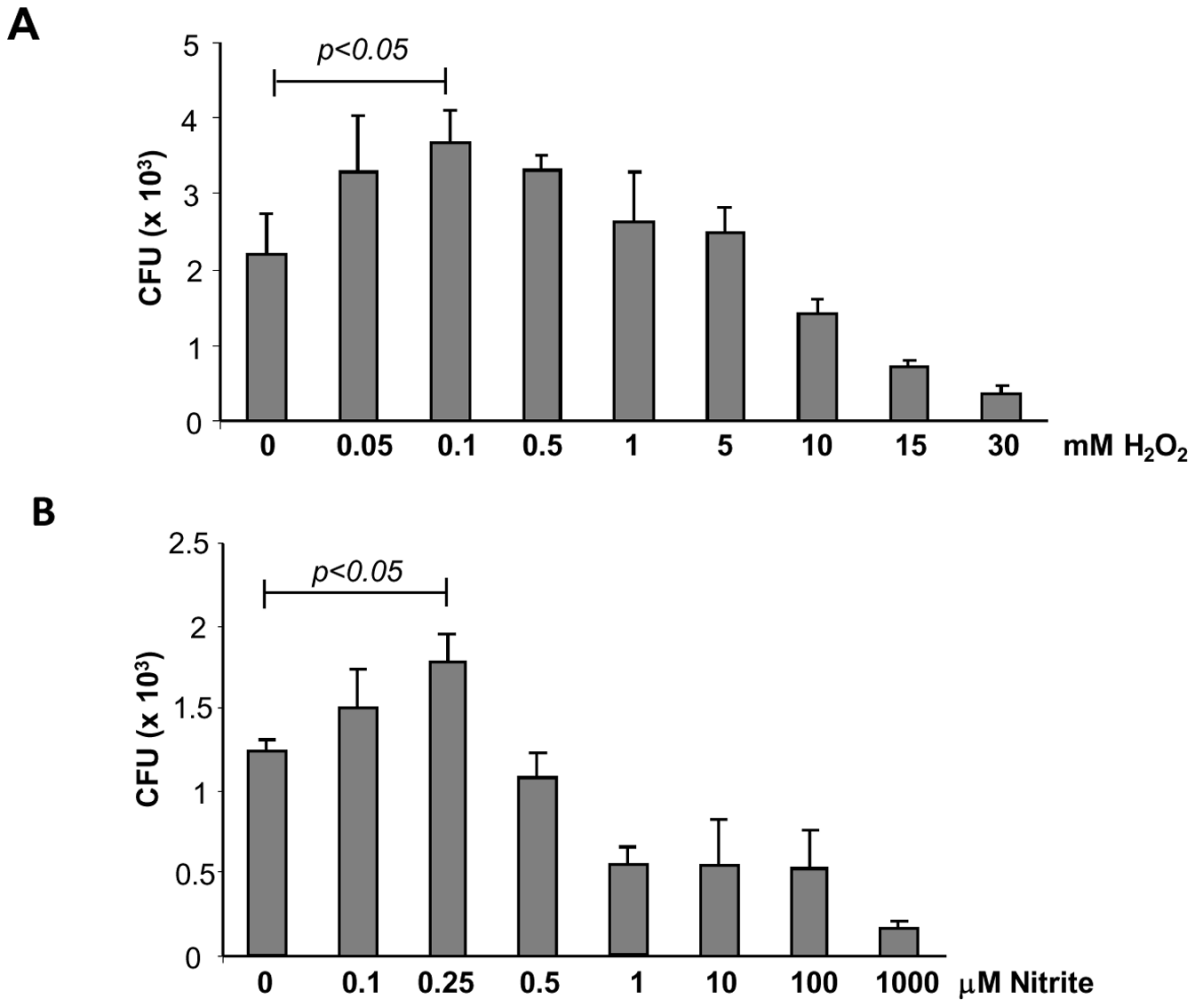
Low concentrations of H_2_O_2_ or nitrite promoted cell proliferation in *P. brasiliensis*. Pb18 yeast cells were seeded in a 6-well culture plate subjected to a 24-h period of starvation with F12 medium and treated with different concentrations of H_2_O_2_ (**A**) or nitrite (**B**) at pH 5.6 for 5 h at 37°C. Treated cells were plated in BHI and incubated at 37°C for 7–10 days (n = 6 at each point). The graph shows the means ± SD of total CFU before and after treatment with H_2_O_2_ or nitrite for each concentration. Statistically significant samples are indicated (*p*<0.05). The results are representative of three independent experiments.

It is known that different levels of H_2_O_2_ or NO can induce distinct responses within a cell [29]. For example, different transcriptional responses are induced by low (sub-toxic) or high (toxic) levels of H_2_O_2_ or NO in mammalian cells, *S. cerevisiae*, and *S. pombe* [30], [31]. To better understand this type of stimulus in *P. brasiliensis*, logarithmic growing yeast cells, were cultured in F12 medium for 24 h and subsequently treated *in vitro* with different concentrations of H_2_O_2_ (0.05–30 mM) and NO_2_ (0.1–1000 µM) for 5 h at 37°C and the CFU count was assessed (Figure 1). For cells pre-incubated for 5 h with higher concentrations of H_2_O_2_ (10, 15 and 30 mM) and NO_2_ (1, 10, 100 and 1000 µM), a typical dose-response curve with decreased cell viability was observed (Figure 1). For intermediate concentrations of H_2_O_2_ (0.5 and 1 mM) and NO_2_ (0.5 µM) there was no change in cell viability (Figure 1). However, yeast cells pre-incubated with low concentrations H_2_O_2_ (0.1 mM) and NO_2_ (0.25 µM) responded with significant cell proliferation (3.7±0.16×10^3^ and 1.8±0.17×10^3^ CFU, respectively) when compared to unstimulated controls (2.13±0.19×10^3^ and 1.35±0.78×10^3^ CFU, respectively). Maximum stimulation of fungal proliferation was observed after incubation with 0.1 mM H_2_O_2_ and 0.25 µM NO_2_. These data suggested that *P. brasiliensis* may benefit from low concentrations of ROS and RNS to proliferate.

### 3.2. Ras participates in P. brasiliensis proliferation dependent on low concentrations of ROS or RNS

**Figure 2:**
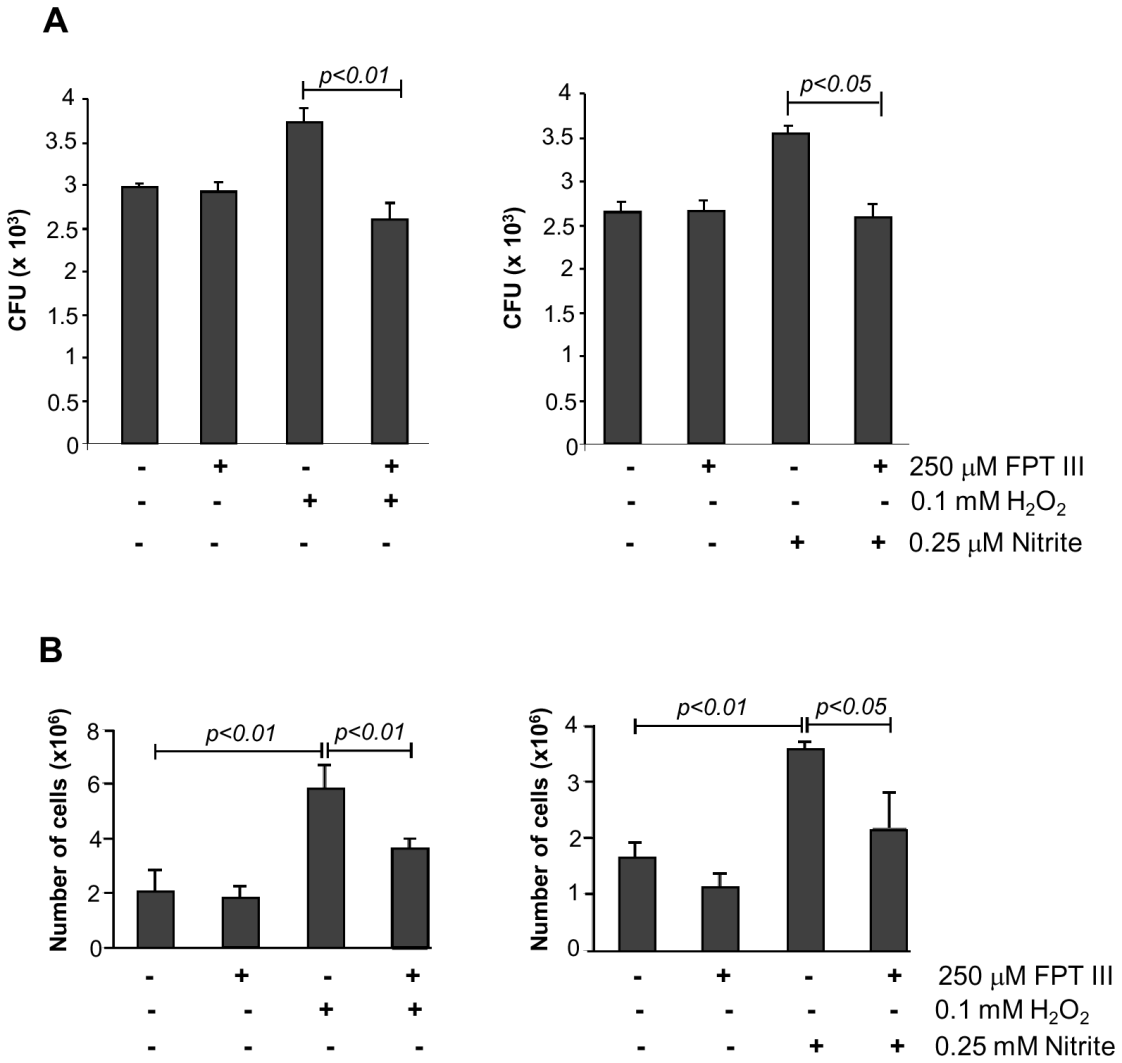
Cell proliferation of *P. brasiliensis* stimulated by low concentrations of H_2_O_2_ or nitrite is suppressed in the presence of FPT III inhibitor. (**A**) Pb18 yeast cells were seeded in a 6-well culture plate subjected to a 24-h period of starvation with F12 medium and pretreated with 250 µM FPT III and stimulated with 0.1 mM H_2_O_2_ or 0.25 µM nitrite at pH 5.6 for 5 h at 37°C. Cells were plated in BHI medium at 37°C for 10 days (n = 6 at each point) and CFU were counted. (**B**) Same as in (**A**), but cells (1.5×10^5^) were cultured in the YPD medium (n = 4 each point) and after 4 days the fungal growth was determined by counting in a Neubauer chamber. The graphs show the mean CFU or number of cells ± SD for each sample. The results represent three independent experiments. Statistically significant samples are indicated (*p*<0.01 or 0.05).

In mammals, one of the major components involved in cell proliferation triggered by ROS and RNS is Ras [32], [33]. Ras must undergo carboxyterminal farnesylation before localizing at the cytoplasmatic side of the plasma membrane [34]. FPT III, a potent and selective inhibitor of the enzyme farnesyl transferase (FTase), efficiently prevents Ras farnesylation [35]. To determine if redox-stimulated cell proliferation is dependent on Ras activation, yeast cells were preincubated for 24-h with FPT III before H_2_O_2_ or NO_2_ exposure. As shown in Figure 2, the growth stimulatory effect with 0.1 mM H_2_O_2_ and 0.25 µM NO_2_ was blocked in presence of this inhibitor (Figure 2A). These results suggested that Ras participates in the cellular proliferation process induced by low concentrations of ROS or RNS.

We also tested the effect of low concentrations of H_2_O_2_ and NO_2_ during fungal growth. *P. brasiliensis* was incubated with or without stimulus in the presence or absence of Ras inhibitor. We observed significant increase in the number of cells growing in the presence of 0.1 mM H_2_O_2_ (5.837±0.825×10^6^ cells) and 0.25 µM of NO_2_ (3.61±0.082×10^6^ cells) after 4 days when compared to controls (2±0.83×10^6^ cells and 1.63±0.288×10^6^ cells, respectively) (Figure 2B), however this effect was abolished in the presence of FPT III. We observed similar results after 8 days of growth (data not shown). However, on the twelfth day there was no statistical difference between the experimental samples and controls (data not shown). Therefore, these results confirm that low concentrations (sub-toxic) of ROS and RNS can lead to *P. brasiliensis* cell proliferation and that Ras farnesylation is required for this event.

### 3.3. Low concentrations of ROS or RNS promote Ras activation in P. brasiliensis

**Figure 3:**
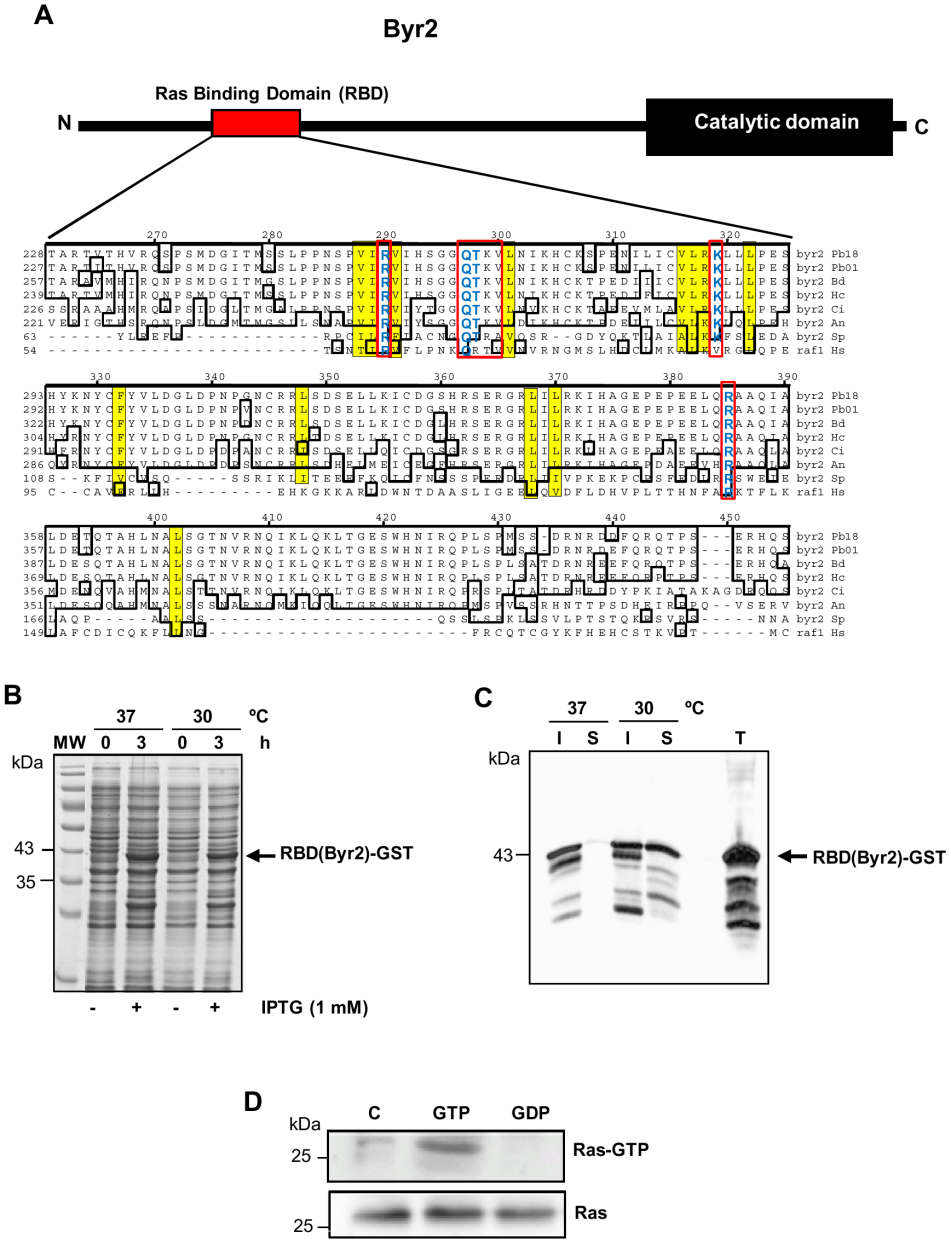
Plasmid construction and production of the RBD(Byr2)-GST probe. (**A**) Schematic representation of Byr2 showing the localization of conserved domains, highlighting in red the localization of the Ras Binding Domain (RBD). RBD sequences from *P. brasiliensis* (Pb – EEH46080), *Blastomyces dermatitidis* (Bd – EGE86103), *H. capsulatum* (Hc – EGC48892), *Coccidioides immitis* (Ci – XP 001242119), *S. cerevisiae* (Sc – AAB67571), and RBD-Raf-1 of *Homo sapiens* (Hs – AGC09606) were aligned with ClustalW (module MegAlign, DNAstar Inc). Conserved sequences are boxed, the residues directly involved in the interaction with activated Ras (Ras-GTP) are indicated in red boxes and key amino acids involved in the interaction with Ras-GTP are shown in yellow. (**B**) Coomassie blue-stained 10% SDS-PAGE gels showing total bacterial extracts from recombinant bacteria expressing RBD(Byr2)-GST (arrow) stimulated (3 h) or not (0 h) with 1 mM IPTG. (**C**) Ten microliters of total (T), soluble (S) and insoluble (I) fractions were assayed in Western blots probed with anti-GST antibody. The migration of molecular mass standards (MW) is shown on the left. (**D**) Pb18 total extract (1 mg) was bound either to GTP (1 mM) or GDP (1 mM) and incubated with RBD(Byr2)-GST fusion protein linked to glutathione-Sepharose. Ras-GTP and total Ras (50 µg total protein) eluted with SDS-PAGE sample buffer were loaded in an SDS-PAGE gel. Ras was detected by immunoblotting with anti-Ras antibody.

To understand the contribution of Ras on *P. brasiliensis* redox-dependent proliferation, we evaluated its activation after stimulation with low concentrations of H_2_O_2_ and NO_2_. In order to do that, we constructed a probe that detects Ras active form (Ras-GTP). In fungi, the serine/threonine kinase Byr2 (Ste11 homologue in *S. cereviseae*) is known to be responsible for the interaction with activated Ras. In *P. brasiliensis*, the Byr2 gene is composed of four exons separated by three introns. *BYR2* has 2,685 bp and encodes a protein of 894 amino acids. The Ras Binding Domain (RBD) shows conserved Ras interaction regions (Figure 3A), as observed in the alignment of Byr2-RBD with its fungal homologues from *A. niger*, *S. cerevisiae* and *S. pombe*, and with human Raf-1. The RBD is located in a regulatory region at the *N*-terminal, between amino acids 216 and 395. According to Scheffzek et al. [36], the Byr2-RBD amino acid residues from *S. pombe* responsible for the interaction with Ras would be Arg74, Lys101, Ala84, Arg83, Thr82, Gln81, and Arg160. All residues are preserved in *P. brasiliensis* Byr2-RBD, suggesting that the intermolecular interaction with activated Ras may also occur (Figure 3A).

The use of a RBD-GST probe in studies on cell signaling is widespread to detect Ras activity in mammalian cells [33], [37], [38]; however, in fungi there are only few studies using this strategy [27], [39], [18]. We constructed an expression plasmid containing approximately 537 pb of the Byr2-RBD gene fragment subcloned into *Eco*RI and *Xho*I sites of the pGEX-4T-2 vector. The cloned fragment corresponds to Byr2 amino acids 216–395 (179 amino acids) that include the whole RBD. For expression of the recombinant protein (RBD-GST probe), the expression vector was introduced into *E. coli* BL21 and induced with 1 mM of IPTG at 37°C or 30°C. After 3 hours of induction in both temperatures a component of approximately 43 kDa was expressed, which is consistent with the expected molecular mass of the recombinant protein (Figure 3B). The solubility of recombinant RBD(Byr2)-GST was evaluated and we observed that at 37°C most of the fusion protein was expressed as insoluble inclusion bodies (Figure 3C). However, at 30°C we observed that a greater proportion of the RBD(Byr2)-GST recombinant protein was found in the bacterial extract soluble fraction (Figure 3C). To determine specificity of the probe RBD(Byr2)-GST in detecting Ras-GTP we performed *in vitro* exchange experiments. We incubated Pb18 total extract with high amounts of GTP or GDP, probed it with RBD(Byr2)-GST and immunoblotted the precipitate with anti-Ras. Only activated Ras (Ras-GTP) was able to bind to the probe RBD(Byr2)-GST (Figure 3D), indicating that the assay is specific for Ras-GTP.

**Figure 4:**
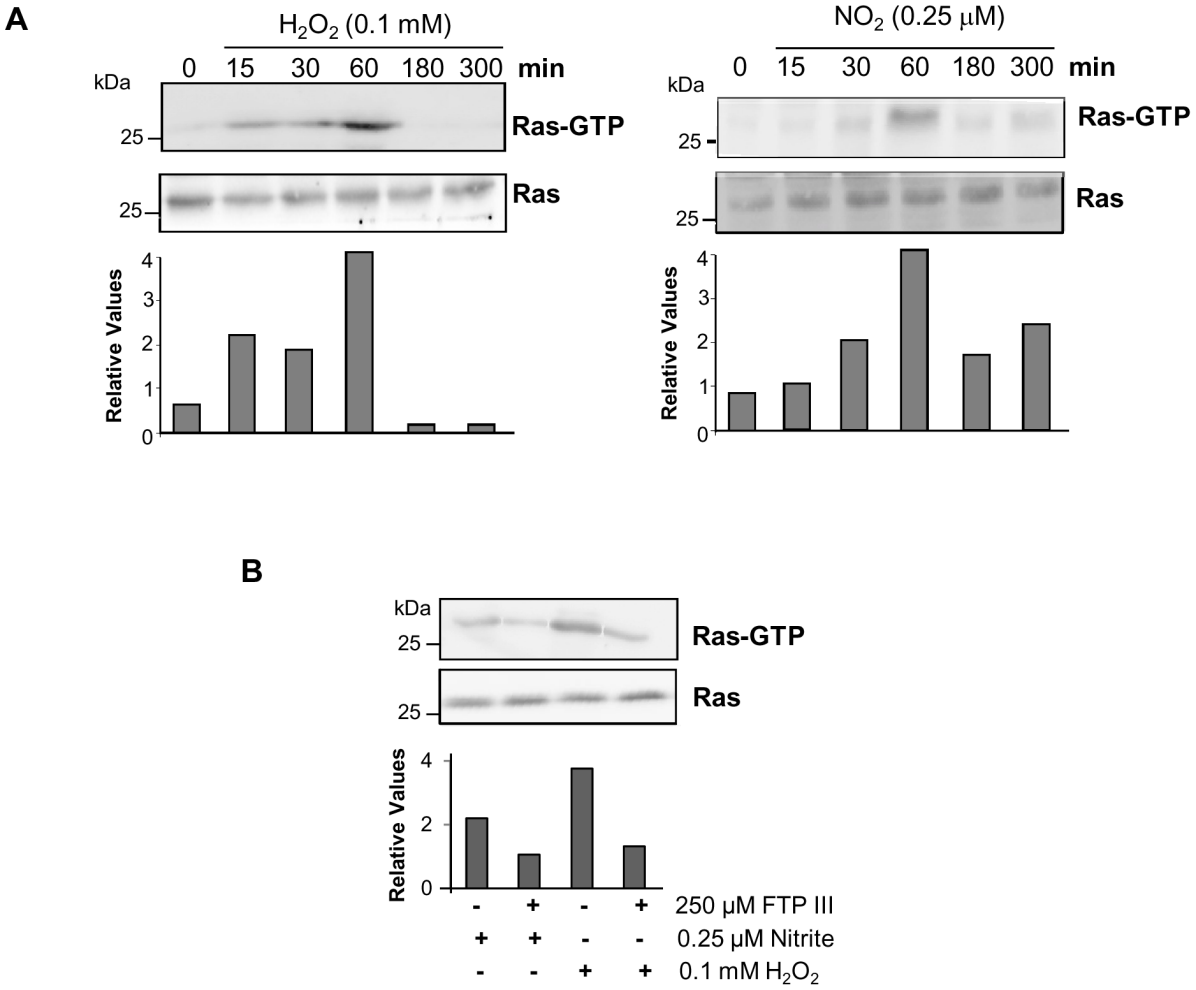
Low concentrations of H_2_O_2_ or nitrite induce Ras activation. (**A**) Pb18 yeast cells were cultivated in modified YPD for 5 days at 37°C, subjected to a 24-h period of starvation with F12 medium and incubated with 0.1 mM H_2_O_2_ or 0.25 µM NO_2_ at pH 5.6 for different timepoints at 37°C. (**B**) Pb18 yeast cells were cultivated in modified YPD for 5 days at 37°C, subjected to a 24-h period of starvation with F12 medium and cultured with or without 250 µM FPT III inhibitor followed by treatment with 0.1 mM H_2_O_2_ or 0.25 µM NO_2_ at pH 5.6 for 1 h at 37°C. After yeast cells lysis (**A**) and (**B**), Ras activation was determined using the GST-RBD(Byr2) fusion protein, which binds with high affinity to the GTP-associated form of Ras. Ras-GTP (active) and total Ras (50 µg total protein) were assayed by western blots probed with anti-Ras antibody. Relative densitometric values of bands are shown in the bar graphs.

We next used the RBD(Byr2)-GST probe to assess the ability of low concentrations of ROS and RNS to activate Ras. Ras activity was determined in *P. brasiliensis* yeasts exposed to 0.1 mM H_2_O_2_ and 0.25 µM NO_2_ for increasing periods of time. Early Ras activation was observed after 15 min of stimulation with ROS and RNS (Figure 4A). Maximum Ras activation was observed after 60 min of incubation (Figure 4A), and at latter timepoints (180 and 300 min) we observed decrease of Ras activation. Also, the fungal protein extract was incubated with Glutathione-Sepharose beads alone (negative control) and analyzed by Western blot, but no reaction was observed (data not shown). The protein loading of the samples used in the Ras activity assay observed in SDS-PAGE gel stained Coomassie blue proved to be fairly homogeneous (data not shown). We also evaluated by RT-PCR the transcription levels of *RAS1* and *RAS2* in *P. brasiliensis* after incubation with 0.1 mM H_2_O_2_ or 0.25 µM NO_2_, but no significant changes were observed (data not shown). Therefore, low concentrations of ROS and RNS promoted guanine nucleotide exchanges in the critical cellular signaling protein Ras.

We also investigated the inhibitory effect of farnesyl transferase in Ras activity. Yeast cells were cultivated for 24 h in the presence or absence of 250 µM FPT III, followed by treatment for 1 h with 0.1 mM H_2_O_2_ or 0.25 µM nitrite. As shown in Figure 4B, FPT III substantially inhibited Ras activation in yeast cells of *P. brasiliensis* treated with low concentration of ROS or RNS, as compared with controls without inhibitor.

### 3.4. NO_2_ promotes Ras S-nitrosylation in P. brasiliensis

**Figure 5:**
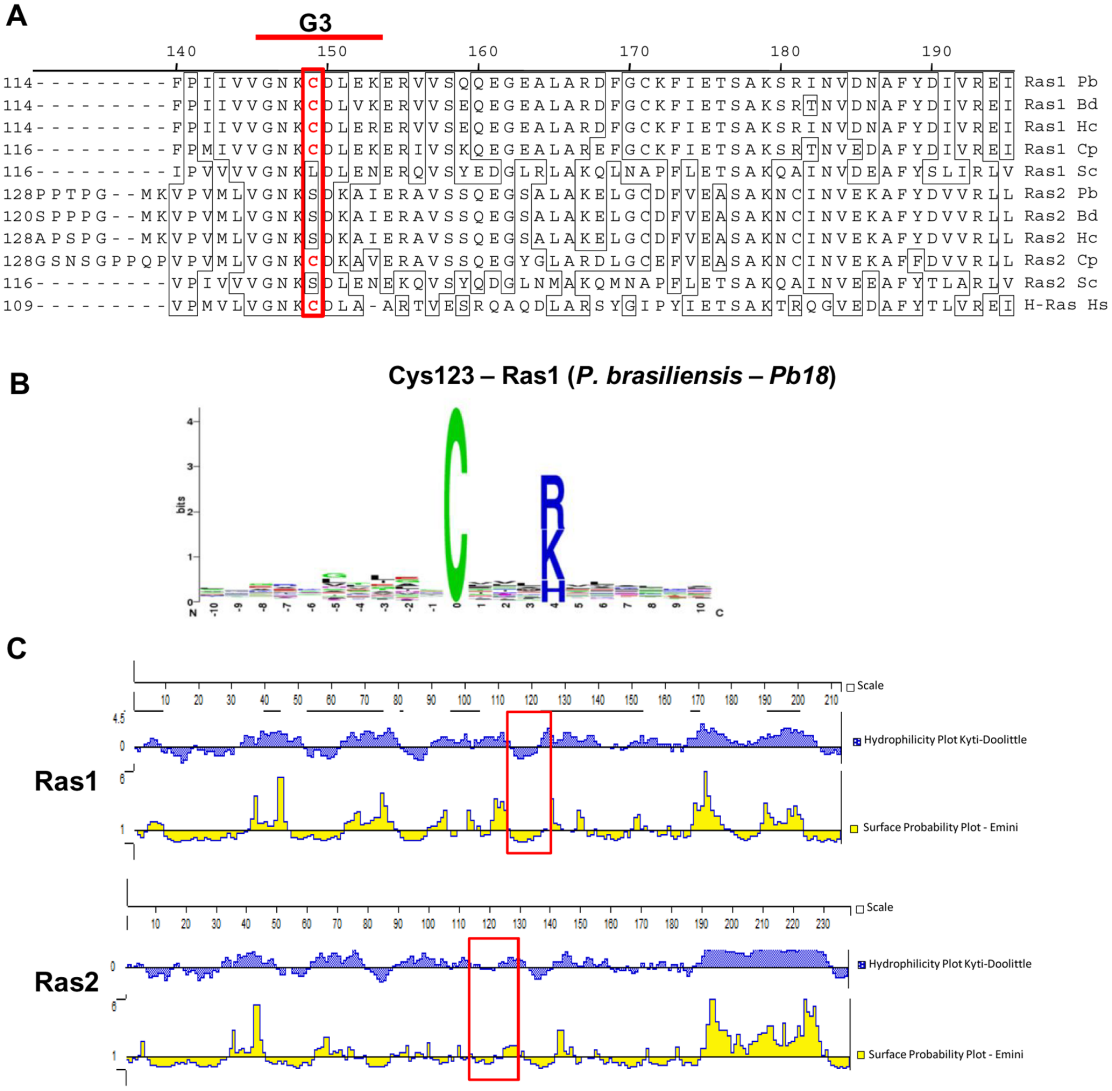
*P. brasiliensis* Ras1 has a putative nitrosilable Cys. (**A**) Ras1 and Ras2 sequences from *P. brasiliensis* (Pb – EEH22637 and EEH22450), *B. dermatitidis* (Bd – XP002628159 and EEQ83443), *H. capsulatum* (Hc – EEH06649 and EEH07767), *C. posadasii* (Cp – XP 001246878 and XP 001247157), *S. cerevisiae* (Sc – AAA34958 and CAA95974) and *H. sapiens* (Hs – AAH14261) were aligned by ClustalW (module MegAlign, DNAstar Inc). (**B**) Analysis of putative motifs of *S*-nitrosylation in Pb18 Ras1 using the computer program dbSNO (http://dbSNO.mbc.nctu.edu.tw, [37]). (**C**) Kyte-Doolittle hydrophilicity and Emini Surface Probability plots (Protean module; DNAstar Inc.) of Pb18 Ras1 and Ras2. Red boxes indicate the location of Ras1 Cys123 and Ras2 Ser144, respectively.

**Figure 6:**
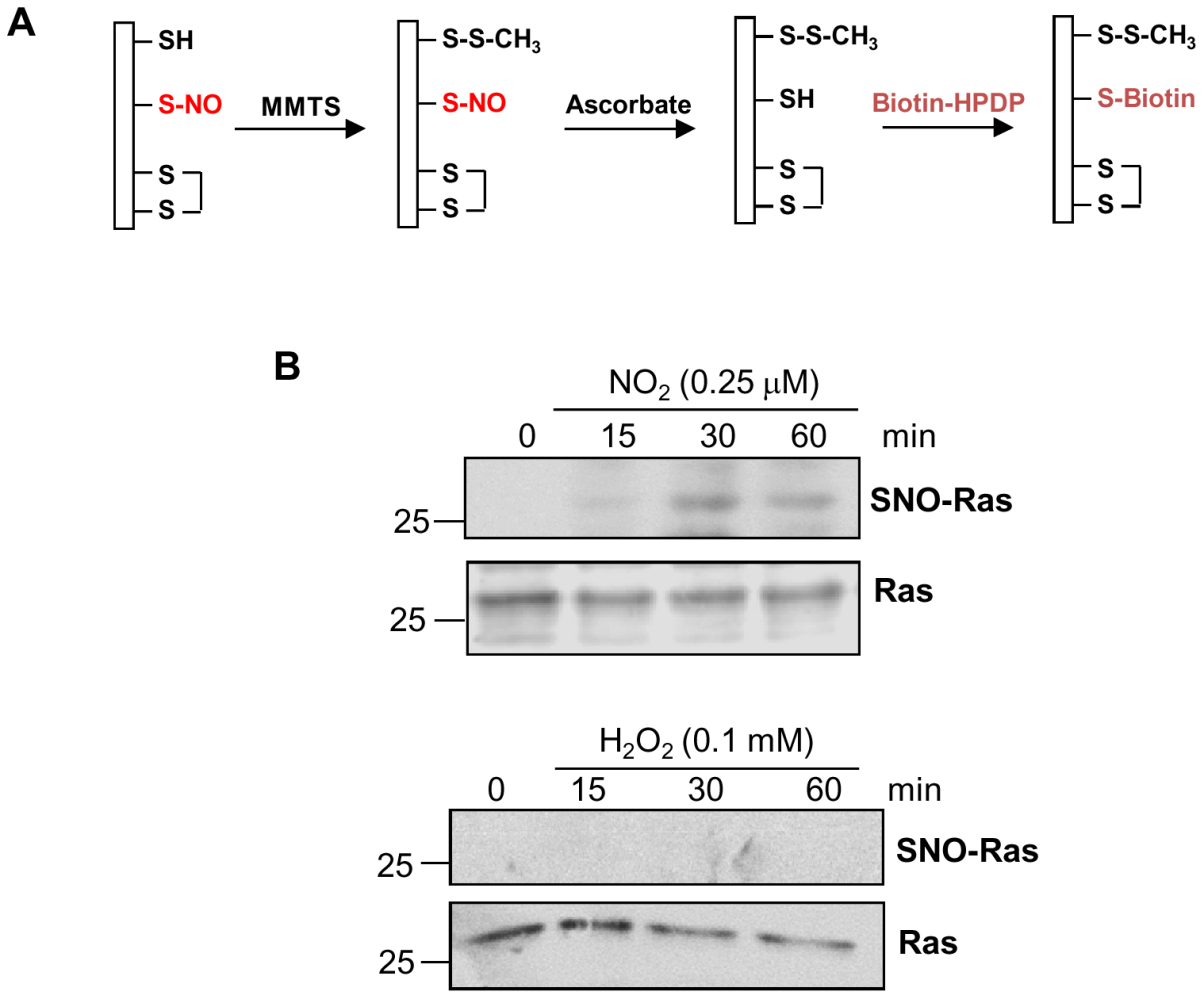
Low concentrations of nitrite induce Ras *S*-nitrosylation in *P. brasiliensis*. (**A**) Schematic diagram of the *S*-nitrosylation assay. A cysteine is indicated with a free thiol, disulfide, or nitrosothiol conformation. The free thiols are made unreactive by methylthiolation with MMTS. Next step, nitrosothiols are selectively reduced with ascorbate to reform the thiol, which then reacts with the thiol-modifying reagent biotin-HPDP. (**B**) *In vitro S*-nitrosylation of *P. brasiliensis* extracts after stimulus with 0.1 mM H_2_O_2_ or 0.25 µM NO_2_ at pH 5.6 for increasing periods at 37°C. Cell extracts were analyzed by the biotin-switch technique (see the Materials and Methods sections for details), and Western blots were probed with anti-Ras antibody.

It has been shown that NO stimulates guanine nucleotide exchange in Ras and that this event is dependent on *S*-nitrosylation, which occurs in the active thiol group Cys118 [40]. Initially we checked whether nitrosylable Cys was conserved in *P. brasiliensis*. By aligning a small fragment of the C-terminal region from different fungi and human Ras, we observed that in *P. brasiliensis* Ras1 there is a Cys homologue at position 123, whereas in Ras2 there is a serine at this position (Figure 5A). Then we used a computer program (http://dbSNO.mbc.nctu.edu.tw, [41]) to predict putative *S*-nitrosylation sites in *P. brasiliensis* Ras and detected that Cys123 would be a likely *S*-nitrosylation site with 95% prediction specificity (Figure 5B). Cys123 is localized in the Ras GTP binding and interaction site (G3) (Figure 5A). Ras2 also showed probable *S*-nitrosylation sites at Cys55 and Cys176; however, these sites are located elsewhere in the molecule (data not shown). We also evaluated whether Cys123 would be located in Ras hydrophobic region. According to Hess et al. [6] local hydrophobicity might promote specific *S*-nitrosylation. Analysis of the deduced *P. brasiliensis* Ras1 and Ras2 sequences showed that Cys123 is inserted in Ras1 hydrophobic domain with low surface-probability in the protein (Figure 5C). Moreover, this feature was not observed in the equivalent Ras2 region. These results suggested that Ras1 Cys123 bears *S*-nitrosylation features. Therefore, we verified if Ras1 was possibly *S*-nitrosylated by the biotin switch test [28], which introduces a biotin molecule in the *S*-nitrosylated Cys (Figure 6A). The detection of *S*-nitrosylated protein by the biotin switch method is dependent on treatment with ascorbate, consistent with the reduction dependent on ascorbate from nitrosothiol bonds (Figure 6A). For that, we studied whether lower concentrations of NO_2_ (0.25 µM) or H_2_O_2_ (0.1 mM) would be able to induce Ras1 *S*-nitrosylation. We found that NO_2_ induced rapid increase in *S*-nitrosylated Ras levels, with a peak at 30 min (Figure 6B). On the other hand, no change in the level of *S*-nitrosylation was observed after treatment with H_2_O_2_ (Figure 6B). These findings suggested that NO probably plays an important role in the process of Ras activation through its *S*-nitrosylation.

## Discussion

The ability of pathogenic fungi to resist the deleterious effects of ROS and/or RNS is foreseen as an important virulence mechanism, particularly in relation to its contact with phagocytic host cells. We have here demonstrated that sub-toxic concentrations of ROS and RNS are capable of stimulating *P. brasiliensis* cell proliferation in a Ras GTPase activation-dependent manner. In addition, we observed that stimulation with NO_2_ evoked Ras *S*-nitrosylation, which has not been observed after stimulation with H_2_O_2_. Cell proliferation upon ROS and RNS stimulation have not been reported before in fungi; however, it remains to be clarified if this phenomenon would occur in *P. brasiliensis* isolates from other phylogenetic groups [42], or even in isolates of the recently separated species *P. lutzii* [43], apart from virulent Pb18 presently tested. Pb18 represents the main S1 phylogenetic *P. brasiliensis* group [42].

It has frequently been observed that high concentrations of both ROS and RNS are cytotoxic to fungal cells, causing cell death, and this is an effector mechanism of the immune system cells [44]. However, NADPH oxidase knockout mice (deficient for ROS production) showed decreased fungal spread when intratracheally infected with *C. neoformans*, and were also protected against pulmonary infection [45]. This event, which seems to contradict the literature, may suggest that the microorganisms could somehow benefit from ROS/RNS derived from immune system cells. Our data suggests that one possible mechanism would be by direct contact of the fungus with sub-toxic concentrations of NO_2_ and H_2_O_2_, but that remains to be tested in *C. neoformans*. Despite the proliferation induced by low doses of ROS and RNS having been previously demonstrated in mammalian cells [32], [33], [46], this is the first report of stimulation of fungal cell growth by ROS/RNS. Recently, Srinivasa et al. [47] showed that both low (<1 mM) and high (4–10 mM) concentrations of exogenous H_2_O_2_ induce filamentous growth with distinct cell morphology and growth rate in *C. albicans*, which suggests a differential transcriptional response. The authors demonstrated that sub-toxic doses of H_2_O_2_ induce the formation of pseudohyphae.

In experimental PCM, NO has dual roles. Brummer et al. [1] have demonstrated that activation of mouse (BALB/c) peritoneal macrophages by IFN-γ enhances the fungicidal activity. In contrast, macrophages from A/J mice were poorly activated by low doses of IFN-γ, and secreted only low amounts of IL-12, NO, thus showing poor fungicidal ability [48]. Nascimento et al. [49] showed that NO is essential for host resistance to infection by *P. brasiliensis*. The authors found that mice genetically deficient for iNOS (inducible nitric oxide synthase) were susceptible to infection by the fungus. On the other hand, the persistent production of NO was correlated with increased infection by *P. brasiliensis* [49]. Our *in vitro* data suggests that one possible alternatively mechanism of fungal proliferation *in vivo* would be by direct contact of the fungus with sub-toxic concentrations of NO_2_ and H_2_O_2_.

In our study we observed that there was decrease in Ras activity after incubation with FPT III (Fig. 4B). Previous results with the *P. brasiliensis* showed that farnesylation blockage interfered with vegetative growth of yeast cells and stimulated germinative tube production even at 37°C [21]. Since the farnesylation inhibitor is not specific for Ras incorporation into the membrane, the effects observed in our work could be result from the inhibition of the other Ras-related proteins that are also farnesylated [50]. However, several studies reported the strong involvement of Ras protein in fungal growth and differentiation after different stimuli [17], [51], [52]. Furthermore, it is known that ROS and RNS are involved in Ras-dependent cell proliferation [32], [33], [46], [53] and fungal conidiation [52]. Thus, it is possible speculate that the FPT III effect on fungal growth and Ras activity were dependent on decreasing Ras activity in the same cell (Figure 4A and 4B).

By using a probe developed to detect active Ras, we also demonstrated that, as in mammal cells, *P. brasiliensis* Ras may participate in cell proliferation in a redox-dependent manner. Thus, GTPase Ras is a highly preserved signaling protein that transmits receptor signals from the cell surface to a variety of effectors, thereby regulating important physiological processes such as growth, morphology, and survival in eukaryotes from yeast to humans. Moreover, it can regulate virulence in human pathogenic fungi [54], [55], [56]. Ras can be activated by different stimuli, and it is known that ROS and RNS can induce this process [33],[46],[53]. It is known, for example, that NO, a free radical with signaling properties [57], stimulates human Ras activity by *S*-nitrosylation of the Cys118 residue [40]. *S*-nitrosylation is a reversible posttranslational modification derived from the interaction of NO with the thiol group of specific cysteines [58]. Redox regulation of Ras GTPases occurs in a redox-active cysteine (X) present in a conserved NKXD motif [59]. This Cys homologue was identified only in *P. brasiliensis* Ras1 (Cys123), which was also identified as a putative *S*-nitrosylation site. GTPases with a redox-sensitive NKCD motif can be activated by NO_2_ and other RIs (reactive intermediates). Several cell-based and *in vitro* studies have shown that NO_2_ reacts with Ras through Cys118 to promote nucleotide exchange and Ras activation [60]. Raines et al. [61] speculated that NO_2_-mediated guanine nucleotide release occurs through a radical propagation mechanism involving Ras thiyl radical conversion to a Ras-GDP guanine radical. The guanine base is particularly sensitive to reaction with free radicals [59] and formation of a guanine radical is likely to alter interactions with Ras, resulting in the release of Ras-bound GDP [62]. Thus, NO can increase Ras downstream signaling through the mitogen-activated kinase pathway [63].

On the basis of the above observations, we believe that low levels of NO could lead to Ras *S*-nitrosylation (probably in residue Cys123), which would lead to exchange of GDP for GTP, and consequent GTPase activation in *P. brasiliensis*. That would trigger the Ras-dependent downstream cell-signalling pathway. Recently, protein *S*-nitrosylation has drawn attention as an event capable of inducing biological responses in microorganisms. Seth et al. [64] demonstrated that the transcription factor OxyR from *E. coli*, which is activated by oxidation under aerobic conditions, can also be activated by *S*-nitrosylation under anaerobic conditions, thus inducing gene expression through alternative PTM.

On the other hand, it is known that oxidative modifications trigged by H_2_O_2_ leads to Ras activation [65], and this radical is involved in Ras signal transduction to the nucleus, mediating Ras-induced cell cycle progression [66], [67]. However, the mechanisms by which ROS carries the Ras signal to the nucleus are still unclear.

Testing Ras activity was only possible because of the use of a RBD(Byr2)-GST probe, which detects Ras active form (Ras-GTP). Byr2 from *P. brasiliensis* (in Pb18 this gene was annotated as a dual-specificity mitogen-activated protein kinase - dSOR1) is also known as Ste11, homologous to mammalian Raf1, and it is a target for regulating Ras [36], [68]. In *S. pombe*, the structure of Ras-Byr2-RBD complex revealed that Byr2-RBD shows essentially the same folding structure as that verified in the RBD of Raf-1 [36]. When comparing the Byr2-RBD region of *P. brasiliensis* with that of *S. pombe*, we observed that all major amino acid residues that participate in the intermolecular interaction with Ras are preserved (Figure 3A). We also observed that the residues of interaction with Byr2 corresponding to amino acids Asp33, Glu37, Asp38, Ser39, Arg41 and Asp54, which were detected by Scheffzek et al. [36], are preserved in the Ras1 and Ras2 from *P. brasiliensis*. Thus, we believe that the Byr2-RBD protein from *P. brasiliensis* would be able to interact with the activated Ras form (Ras1 and Ras2); that was actually confirmed by pull-down testing. Our probe has become an important tool to verify Ras activity and it may be further used in other conditions and even with other fungal systems.

In summary, this study demonstrated that in *P. brasiliensis* low concentrations of ROS and RNS can switch the Ras-GDP for Ras-GTP with consequent activation and triggering of a mitogenic signal transduction. This event is interesting because it has been shown that resistance to ROS and RNS is an important virulence factor in pathogenic fungi. In our model, the fungus used sub-toxic concentrations of ROS and RNS to proliferate. In these conditions, in addition to survive, we believe the fungus would grow and develop within low ROS-producer macrophages. It has been proposed that the intracellular parasitism would be an important event for the establishment and progression of PCM in a susceptible host [69]. Thus, a more detailed characterization of these signaling cascades that allow microorganisms to cause disease would be crucial to understand fungal pathogenesis.
